# Comparative effectiveness of non- pharmacological treatments in patients with persistent postural-perceptual dizziness: a systematic review and effect sizes analyses

**DOI:** 10.3389/fneur.2024.1426566

**Published:** 2024-07-12

**Authors:** Zorica Suica, Frank Behrendt, Carina Ziller, Szabina Gäumann, Stefan Schädler, Roger Hilfiker, Katrin Parmar, Hans Ulrich Gerth, Leo H. Bonati, Corina Schuster-Amft

**Affiliations:** ^1^Research Department, Reha Rheinfelden, Rheinfelden, Switzerland; ^2^School of Engineering and Computer Science, Bern University of Applied Sciences, Burgdorf, Switzerland; ^3^Private Practice, Schloss, Sumiswald, Switzerland; ^4^Physiotherapie Tschopp & Hilfiker, Glis, Switzerland; ^5^Translational Imaging in Neurology (ThINk) Basel, Departments of Head, Spine and Neuromedicine and Biomedical Engineering, University Hospital Basel and University of Basel, Basel, Switzerland; ^6^Department of Medicine, University Hospital Münster, Münster, Germany; ^7^Stroke Center and Department of Neurology, University Hospital Basel, Basel, Switzerland; ^8^Department of Clinical Research, University of Basel, Basel, Switzerland; ^9^Department for Sport, Exercise, and Health, University of Basel, Basel, Switzerland

**Keywords:** persistent postural-perceptual dizziness, functional dizziness, non-pharmacological therapy, phobic postural vertigo, visual vertigo

## Abstract

**Introduction:**

The patho-psychological mechanisms of persistent postural-perceptual dizziness (PPPD) appear to be very complex, and a multimodal, multidisciplinary approach is suggested for treating patients with PPPD. The aim of this review was to provide a comprehensive overview of non-pharmacological treatments and their comparative effectiveness in patients with PPPD.

**Methods:**

Scopus, Web of Science, PsycINFO, Medline, Embase, CINAHL, Cochrane Library and ClinicalTrials.gov were searched in April 2022 with a search update in August 2023. Only randomized controlled trials (RCTs) were included. There was no restrictions regarding publication date. Two reviewers independently identified eligible trials, extracted data, double-checked all extracted information from the included articles and assessed the risk of bias using the Cochrane risk of bias tool. A qualitative synthesis was performed, considering methodological heterogeneity between trials. Finally, an effect size analysis was performed for each treatment comparison. The standardized mean differences (SMD) and their corresponding 95% confidence intervals (95%CI) were calculated for each trial using Review Manager 5.4.

**Results:**

Thirteen RCTs (618 patients with moderate or mild dizziness) out of 1,362 references describing seven different non-pharmacological comparisons were selected. Nine trials included patients with PPPD, and four trials included patients with functional dizziness. The trials used different interventions that were classified as: (1) psychotherapeutic interventions (cognitive behavioral therapy, patient education), (2) physiotherapeutic interventions/training (vestibular rehabilitation, optokinetic stimulation), (3) stimulation procedures (vagus nerve stimulation, transcranial direct current stimulation) and (4) device application (visual desensitization using personalized glasses). However, most of the trials investigated the effects of single interventions, rather than multimodal interdisciplinary treatment of patients with PPPD. The SMD for dizziness handicap and severity was between 0.04 and 0.52 in most trials. In one trial using visual desensitization, the SMD was 1.09 (strong effect on the severity of dizziness) and 1.05 (strong effect on dizziness handicap).

**Discussion:**

Several individual interventions have shown benefits in the treatment of patients with PPPD with small to moderate effects. However, the multimodal treatment or a combination of vestibular rehabilitation with visual desensitization, cognitive behavioral therapy including patient education, and medication support should be further investigated. Future trials should include a large sample size with severe dizziness, and provide a longer follow-up period.

**Clinical trial registration:**

PROSPERO CRD42022320344.

## 1 Introduction

Persistent postural-perceptual dizziness (PPPD) is a chronic disorder of the nervous system, manifested by one or more symptoms of dizziness, unsteadiness, or non-spinning vertigo, present on most days for 3 months or more (1). Symptoms may be exacerbated by upright posture, active or passive movement, and exposure to moving or complex visual stimuli [see Table 1 for diagnostic criteria of the Bárány Society by Staab et al. (1)].

**Table 1:** Bárány Society criteria for the diagnosis of persistent postural-perceptual dizziness (PPPD) by Staab et al. (1).

**A. One or more symptoms of dizziness, unsteadiness, or non-spinning vertigo are present on most days for 3 months and more**
1. Symptoms are persistent, but wax and wane
2. Symptoms tend to increase as the day processes but may not be active throughout the entire day
3. Momentary flares may occur spontaneously or with sudden movements
**B. Symptoms are present without specific provocation but are exacerbated by**
1. Upright posture
2. Active or passive motion without regard to direction or position
3. Exposure to moving visual stimuli or complex visual patterns
**C. The disorder usually begins shortly after an event that causes acute vestibular symptoms or problems with balance, though less commonly, it develops slowly**
1. Precipitating events include acute, episodic, or chronic vestibular syndromes, other neurologic or medical illnesses, and psychologicaldistress
a. When triggered by an acute or episodic precipitant, symptoms typically settle into the pattern of criterion A as the precipitant resolves, but may occur intermittently at first, and then consolidate into a persistent course
b. When triggered by a chronic precipitant, symptoms may develop slowly and worsen gradually
**D. Symptoms cause significant distress or functional impairment**
**E. Symptoms are not better attributed to another disease or disorder**

PPPD is one of the most common causes of chronic dizziness, with a reported prevalence of up to 20% in middle-aged patients (2–4), and with a significant functional impairment, and therefore high impact on quality of life (5, 6). In addition, patients with PPPD also have an increased risk of anxiety and depression (5).

Although the term PPPD is relatively new, the disorder is not, and its main features have been described for at least three decades using synonyms like phobic postural vertigo (PPV), chronic subjective dizziness (CSD), space-motion discomfort (SMD) and visual vertigo (1, 7). These synonyms are included in the International Classification of Diseases (11th Revision, ICD-11) beta draft definition of PPPD, as endorsed by the World Health Organization (WHO) (7). Furthermore, the term ‘functional vertigo and dizziness’ has been defined by Dieterich and Staab as a new nomenclature to refer to one and the same construct, which had previously been given very different terms, such as somatoform dizziness, phobic postural dizziness, visual vertigo, or persistent postural perceptual dizziness (7).

PPPD could be initially triggered by disorders such as vestibular neuritis, benign paroxysmal positional vertigo (BPPV), Meniére’s disease, vestibular migraine, stroke or panic attacks. All triggers have in common that the vestibular system is inhibited, leading to the dominance of the somatosensory and visual systems (1, 8). Nevertheless, the results of the physical examination, vestibular evaluation, and clinical laboratory tests may be normal in patients with PPPD (1). Two systematic reviews, that included only neuroimaging studies of patients with PPPD, found evidence for a reduction of cortical folding and grey matter volume in the multisensory vestibular cortex, visual cortex, cerebellum, and prefrontal and emotional regulatory areas. In addition to the structural changes, abnormal activation and connectivity in the vestibular cortex, in particular the parieto-insular vestibular cortex (PIVC), the visual cortex, the cerebellum and the anxiety-related network in patients with PPPD were also observed (9, 10). Together, these neuroimaging findings may explain the core symptoms of PPPD, such as postural unsteadiness and visually induced dizziness.

Although the pathogenesis of PPPD needs to be further elucidated and understood, several previous studies have described three key mechanisms that may explain the pathogenesis of PPPD (7, 11). These include stiffened postural control, a shift in the processing of perception and orientation of the surrounding environment to favor visual over vestibular inputs, and a failure of the higher cortical mechanisms to modulate the first two processes. In healthy individuals, the normal physiological response to the onset of dizziness, vertigo or the risk of falling is to activate a high-risk postural control strategies, such as a stiffened stance or shorter strides and to rely more on visual or somatosensory inputs than on vestibular signals. These strategies are abandoned when the postural threat subsides. In contrast, patients with PPPD are likely to persistently maintain a high-risk postural control strategy, excessive vigilance for dizziness and imbalance, and visual dependence for perception and orientation of the surrounding environment even when the threat subsides. Neurotic personality traits and pre-existing anxiety predispose patients to failure of re-adaptation (1, 7, 11, 12).

In view of such complex patho-psychological mechanisms, there is no single method for the treatment of PPPD. Therefore, it is proposed to use a multimodal, multidisciplinary approach to treat PPPD (7, 12, 13).

In their narrative review, Sun and Xiang (14) discussed possible non-pharmacological treatment options for patients with PPPD, such as vestibular rehabilitation, cognitive behavioral therapy, and vagus nerve stimulation. Moreover, the vestibular rehabilitation based on evidence-based Clinical Practice Guidelines is considered to be the ‘gold standard’ for the treatment of patients with impaired balance control in various vestibular and neurological disorders (15), and is also recommended for patients with PPPD (12).

To date, there is no systematic overview and meta-analysis of the different non-pharmacological treatments used in patients with PPPD. In fact, the recently published Cochrane review focused on the non-pharmacological treatment, but the selection criteria limited the interventions evaluated to talking therapies or stress management, vestibular rehabilitation, or transcranial direct current stimulation with a follow-up assessment of more than 3 months (16).

The aim of our systematic review and the effect size analyses was to provide a comprehensive overview of non-pharmacological treatments and to compare their effectiveness for patients with PPPD, without restriction regarding the length of the follow-up period. This should help clinicians to choose an appropriate intervention according to the agreed rehabilitation goals.

## 2 Methods

The protocol for this review was registered in the International Prospective Register of Systematic Reviews (PROSPERO; https://www.crd.york.ac.uk/prospero/), registration number CRD42022320344. Further, we used the Preferred Reporting Items for Systematic review and Meta-Analysis (PRISMA) guidelines and the PRISMA checklist to conduct and report this systematic review (17).

### 2.1 Search strategy and selection of trials

A professional librarian at the University of Zurich (CH) searched the following seven databases since their inception until April 2022: Scopus, Web of Science, PsycINFO, Medline, Embase, CINAHL, Cochrane Library, and ClinicalTrials.gov. An update of the search in all databases based on the previous professional search was performed by the first author in August 2023. Search terms, and selection criteria were based on the PICOS system (Table 2). The PRISMA-S checklist was used to report the literature search (18).

**Table 2:** Inclusion criteria.

Population	Patients with persistent postural–perceptual dizziness (PPPD)
Intervention	All treatments of PPPD, such: vestibular rehabilitation, optokinetic stimulation, virtual reality, noninvasive brain stimulation (i.e. transcranial direct current stimulation, transcranial alternating current stimulation), psychotherapy, cognitive behavioral therapy etc.
Compare	Any non-pharmacological interventions, or no therapy
Outcome	(1) Dizziness handicap and severity of dizziness measured with patient-reported outcome measures; (2) Balance and gait; (3) Quality of life
Study design	Controlled trials, randomised controlled trials

The search strategy was adapted for each database and included combined terms regarding the population and trial design:

- (Persistent postural–perceptual dizziness OR space-motion discomfort OR phobic postural vertigo OR visual vertigo OR chronic subjective dizziness OR functional dizziness OR visually induced dizziness OR visual dependence OR somatoform vertigo) AND (clinical trial OR randomized controlled trial) (Supplementary file S1).

Articles were excluded if the authors described surgical procedures or a pharmacological treatment only. Other forms of vertigo as primary diagnosis and with symptoms present for at less than 3 months were excluded: [i.e., vestibular hypofunction (unilateral/bilateral), benign paroxysmal positional vertigo (BPPV), Meniere’s disease, vestibular migraines, canal dehiscence, acoustic neuroma (vestibular schwannoma), acute dizziness and cervicogenic dizziness].

All references were imported into the reference management software package, EndNote (version X7; Thomson Reuters, New York, United States). De-duplication was performed by the university librarian, who conducted the original search. The Covidence software package was used for reference screening (19). Two out of three reviewers (ZSU, CZ, and SG) independently screened all titles, abstracts, and full texts of the identified trials. If no full text was available, the corresponding authors of the articles were contacted to obtain the missing papers. Disagreements between the reviewers were resolved by consulting an independent consensus reviewer (CSA). Finally, the reference lists of the included full-text articles were screened for additional references. The Cohen’s kappa statistic and the percentage of inter-rater agreement were calculated to assess the reviewer agreement. Landis and Koch (20) recommend the following classification: poor (0), slight (0.0 to 0.20), fair (0.21 to 0.40), moderate (0.41 to 0.60), substantial (0.61 to 0.80), and almost perfect (0.81 to 1.0).

### 2.2 Data extraction and risk of bias

Two reviewers (ZSU, DS) independently extracted the data, and AB checked all data for accuracy. In the case of inconclusive data (e.g., only graphical presentation, missing variance of change), the original authors or institutions were contacted to obtain the missing information. The following data were extracted:

- Trial- and participant-related information: author, year, country, sample size and study groups, age, and gender of participants, participants’ description
- Trial methodological-related information: kind of randomization, blinding, measurement events, number of drop outs, participants flow chart
- Intervention-related information: outcomes, outcome measures, training content, training duration, training intensity, trial results

The risk of bias of individual trials independently assessed by three reviewers (ZSU, SG, and SD) using the second version of the Cochrane risk-of-bias tool for randomized trials (RoB 2) (21). Five bias domains were rated: (1) Randomization process, (2) Deviations from intended interventions, (3) Missing outcome data, (4) Measurement of the outcome, and (5) Selection of the reported result. The judgments of each domain is included in the overall risk-of-bias judgment, which corresponds to the highest risk of bias in any of the domains (low, high, some concerns).

### 2.3 Data synthesis

Firstly, a qualitative synthesis was performed, considering methodological heterogeneity between trials (i.e., the variation between interventions or comparison on effect modifiers). We were not able to perform pairwise meta-analyses for direct comparison of interventions, as no more than one trial was available for each intervention comparison. However, the standardized mean differences (SMD) and their corresponding 95% confidence intervals (95%CI) were calculated for each trial using Review Manager 5.4 (22). Results are presented as forest plots to visualize the effects of intervention of all primary outcomes.

### 2.4 Primary outcomes

This review focused on the following primary outcomes:

(1) dizziness handicap and severity of dizziness as measured by patient-reported outcome measures; (2) balance and gait; and (3) quality of life.

## 3 Results

**Figure 1:**
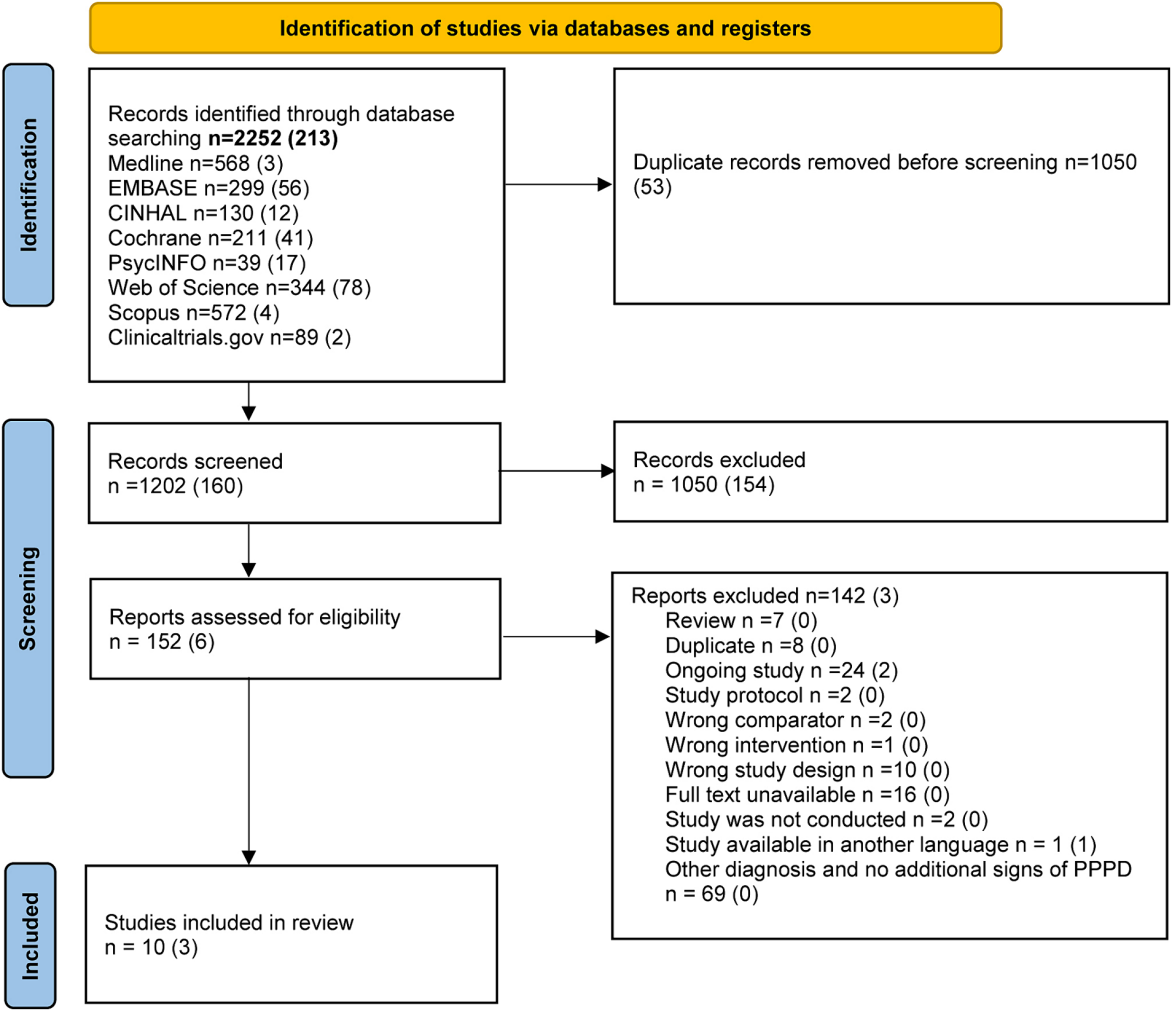
The literature search and trial selection process.

A total of 1,220 references were retrieved in April 2022. The search update in August 2023 resulted in 183 additional references. At the end of the selection process, the kappa was 0.71 (substantial) and the percentage agreement between the raters was 99%. As the Bárány Society and the WHO have defined the PPPD as a new term for functional dizziness, but based on the core features previously researched and described in syndromes such as somatoform dizziness, phobic postural vertigo, or chronic subjective dizziness, we included all trials reporting one of these terms. Nevertheless, we have described and evaluated all trials separately. Finally, 13 trials could be included in our systematic review. Figure 1 shows the process of trial selection and the reasons for exclusion in the full text screening.

Nine trials (23–31) included patients with PPPD, clearly describing the criteria for PPPD as defined by Staab et al. (1). The other four trials (32–36) included patients with functional dizziness, i.e., phobic postural vertigo, somatoform dizziness and visual vertigo.

Different interventions and comparators were reported in the trials, resulting in seven different interventions that were classified into four different categories: (1) psychotherapeutic interventions (cognitive behavioral therapy, patient education), (2) physiotherapeutic interventions/training (vestibular rehabilitation, optokinetic stimulation), (3) stimulation procedures (vagus nerve stimulation, transcranial direct current stimulation) and (4) device application (visual desensitization using personalized glasses, SpotOn Specs).

### 3.1 Description of the trial interventions

#### 3.1.1 Vestibular exercises

Herdman et al. (26) used a customized exercise programme for vestibular rehabilitation: i.e. general exercises involving walking and specific adaptation habituation, visual desensitization, static and dynamic balance exercises. No detailed description of the exercises or the mentioned adaptation is available in their report.

Nada et al. (27) used two types of exercises: gaze stabilization and walking. Gaze stabilization training started with horizontal and then vertical angular head rotations or linear head movements performed while visually fixating a target. Walking exercises started on flat surfaces, forward and backward, and then continued on uneven surfaces, such as a thick carpet. The level of difficulty was subsequently increased by adding head rotations in the form of right and left head-shaking while walking on a hard surface. Other examples included walking backwards and moving over and around obstacles (e.g., in a circle around a table followed by changing the direction of gaze).

Teh et al. (29) performed vestibular exercises programme at home (Bal Ex) and in the clinic. The Bal Ex programme consists of 20 movements divided into 3 levels: (1) head, neck, and eye movements; (2) focus on positioning, movements related to daily activities such as getting up and prayer motion; and (3) work on posture and gait. The step by step approach was to start with 10 repetitions with a slow execution of each movement with an increase to 20 repetitions and a faster execution. Vestibular exercise performed in the clinic mainly consisted of Cawthorne-Cooksey exercises.

Holmberg et al. (32) evaluated the following exercise programme: (1) 15 horizontal head rotations repeated three times with visual fixation of a stationary object while sitting; (2) same procedure but with vertical head movements, (3) horizontal and vertical head movements repeated gradually under successively harder conditions such as standing, standing on a pillow and walking.

#### 3.1.2 Vestibular exercises using virtual reality

Choi et al. (23) performed the vestibular exercises using virtual reality OCULUS Go headset and controller (manufacturer’s information were not included in the original paper). The following vestibular exercise protocol was performed: (1) vestibulo-ocular reflex exercise: the patients rotated their head 10 times for 15° around the target on the centre along the pitch and yaw axis; (2) visual guided vestibulo-ocular reflex exercise: the patients followed the target with their head and eyes. The target moved 15° horizontally and vertically; (3) active head and eye exercise: the patients rotate their head and shift their gaze rapidly to catch-up to the target (spacecraft), which randomly appeared every 10 s and moved around within a 270° visual field.

Yamaguchi et al. (30) applied mediVR KAGURA-guided, dual-task balance training (mediVR KAGURA, Inc., Toyonaka, Japan) in the 3D virtual space in a sitting position. The participants were instructed to catch falling red or blue objects or touch fixed red or blue targets with their right- or left-hand controllers, which takes about 10 min (100 repetitions). Various parameters could be used to determine the level of difficulty, such as distance, angle, height, and size of the object, size of the controller, inter-task interval, falling speed or a time limit for each task.

#### 3.1.3 Optokinetic stimulation

Choi et al. (23) developed an optokinetic stimulation with virtual reality. The device used is not yet commercially available. The patients were asked to watch stars in the virtual night sky, rotating counter-clockwise along the pitch, yaw, and roll axis of their head. Frequency of head movement was 5–10° per seconds over 9 min. Mandour et al. (35) did not provide any information about the therapy content. Only speed used by the optokinetic stimulation and sessions duration were reported.

#### 3.1.4 Vagus stimulation

Eren et al. (24) used the gammaCore® (ElectroCore®, Basking Ridge, United States) device for the non-invasive vagus nerve stimulation. A low-voltage electrical signal were applied [5-kHz sine wave series that occurred for 1 ms and repeated every 40 ms (25 Hz)]. In the phase of an acute exacerbation of dizziness, gammaCore® stimulation was applied on the right side of the neck (right vagus nerve) three times with a stimulation duration of 90 s each. Prophylactic stimulation independent of exacerbations was applied twice a day (i.e., in the morning and in the evening) with also three stimulations of 90 s.

#### 3.1.5 SpotOn specs: active specs and sham specs

Gordon et al. (25) used an interactive software to determine the place and shape of the active markers (Neuro Balance Active Marks (NBA Marks), SpotOn Therapeutics Ltd., Tel Aviv, Israel) attached to eyeglasses. First, visual perception and orientation capacity were assessed using a computer-based continuous performance test. After identifying the active peripheral zones, the NBA marks were applied to the lenses, resulting in the personalized glasses (SpotOn Specs). These marks amplify information about actual head movement, counteracting the mismatch of sensory and motor systems, which should reduce dizziness. Finally, patients were asked to wear the glasses throughout the day for 4 weeks.

In the Sham-specs, the marks were placed in peripheral zones that had previously been classified as neutral and were thought to have no effect on dizziness.

#### 3.1.6 Cognitive behavioral therapy (CBT) and patient education

Herdman et al. (26) applied individual CBT starting with a cognitive behavioral formulation and psychoeducation. Exercises were customized and focussed on normalising any maladaptive postural strategies early on, and on habituation. Other techniques included goal setting, activity planning and graded exercise, attention allocation and relaxation techniques, while the cognitive therapy focussed on illness beliefs, exposure *in-vivo* with behavioral experiments for dizziness related fear, relapse management and prevention. Unfortunately, the original report does not provide any further description.

The main components of CBT in the trial by Yu et al. (28) included: (1) earning the trust of patients; (2) encouraging the patients to communicate with others; (3) making patients expose and check the social factors that cause the PPPD, such as family, work, and social intercourse, and (4) making patients have a correct understanding of the occurrence, development, and treatment of PPPD.

Holmberg et al. (32) informed the patients in one-to-one setting about the nature of CBT and the patients were trained to observe themselves by making written notes about the causes of dizziness, eliciting emotional, cognitive and behavioral responses. Finally, the effects of avoidance behavior on the assessment of threats and the principles of exposure were explained. Methods were developed to counteract the misinterpretation of spontaneous body sway as a sign of imbalance. The patients were educated about the natural process of body sway and the psychologist showed them these movements. It was also suggested to the patients to ask their relatives to assess their postural control and to look in the mirror for feedback. In this way, the patients’ fear of falling and social embarrassment could be reassessed.

Limburg et al. (33) applied a group intervention, where the patients were informed about the psychophysiology of dizziness, dysfunctional cognitions and avoidance behavior. Subsequently, the training sessions were structured as follows: (1) elaboration of individual therapy goals; (2) clarification of interpersonal symptom contexts and accompanying symptoms; (3) differentiation of emotions and body feelings; (4) improvement of self-regulation; (5) symptom-oriented modules focusing on dysfunctional cognitive and interactional patterns; (6) tailored modules focusing on anxiety/phobic, somatoform, and depressive symptoms; (7) transfer to everyday life.

Tschan et al. (36) combined CBT with balance using visual stimuli, and balance board, and relaxation exercises, i.e., breathing exercises. However, CBT included attention focusing, cognitive restructuring, reduction of avoidance behavior, and self-management.

#### 3.1.7 Transcranial direct current stimulation

Im et al. (31) applied transcranial direct current stimulation (tDCS) via two surface electrodes using the YDS-301 N device (YBrain Inc., South Korea). The anodal electrode was placed over the left dorsolateral prefrontal cortex and the cathodal electrode over the right. For the active condition, the current was ramped up to 2.0 mA (current density, 0.07 mA) over 30 s, remained at 2.0 mA for 19 min, and ramped down to 0 mA over 30 s. In the sham group, the current was ramped up to 2 mA over 30 s and ramped down again over the next 30 s.

### 3.2 Risk of bias within trials

**Figure 2:**
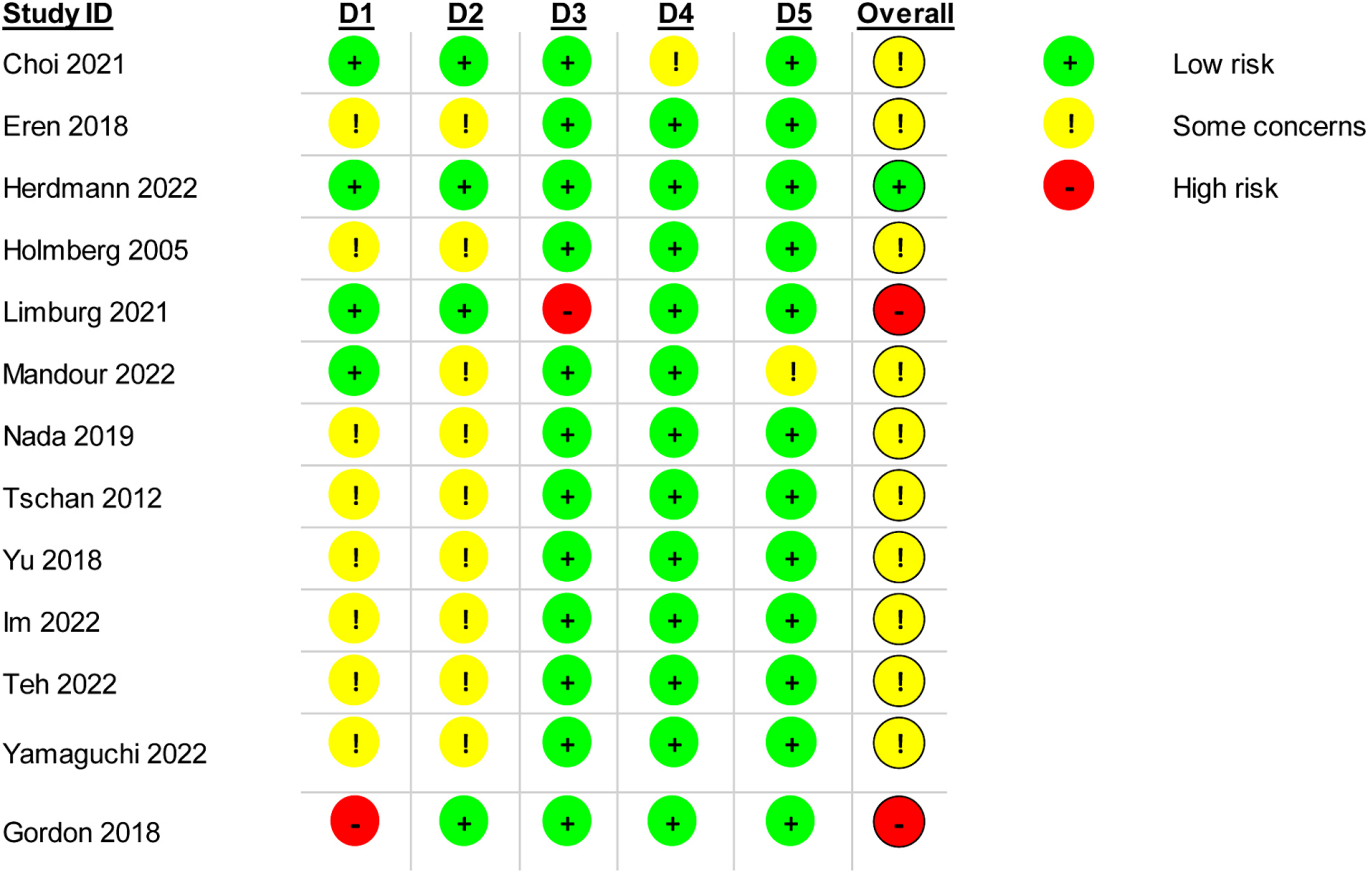
Risk of bias rating for each trial.

**Figure 3:**
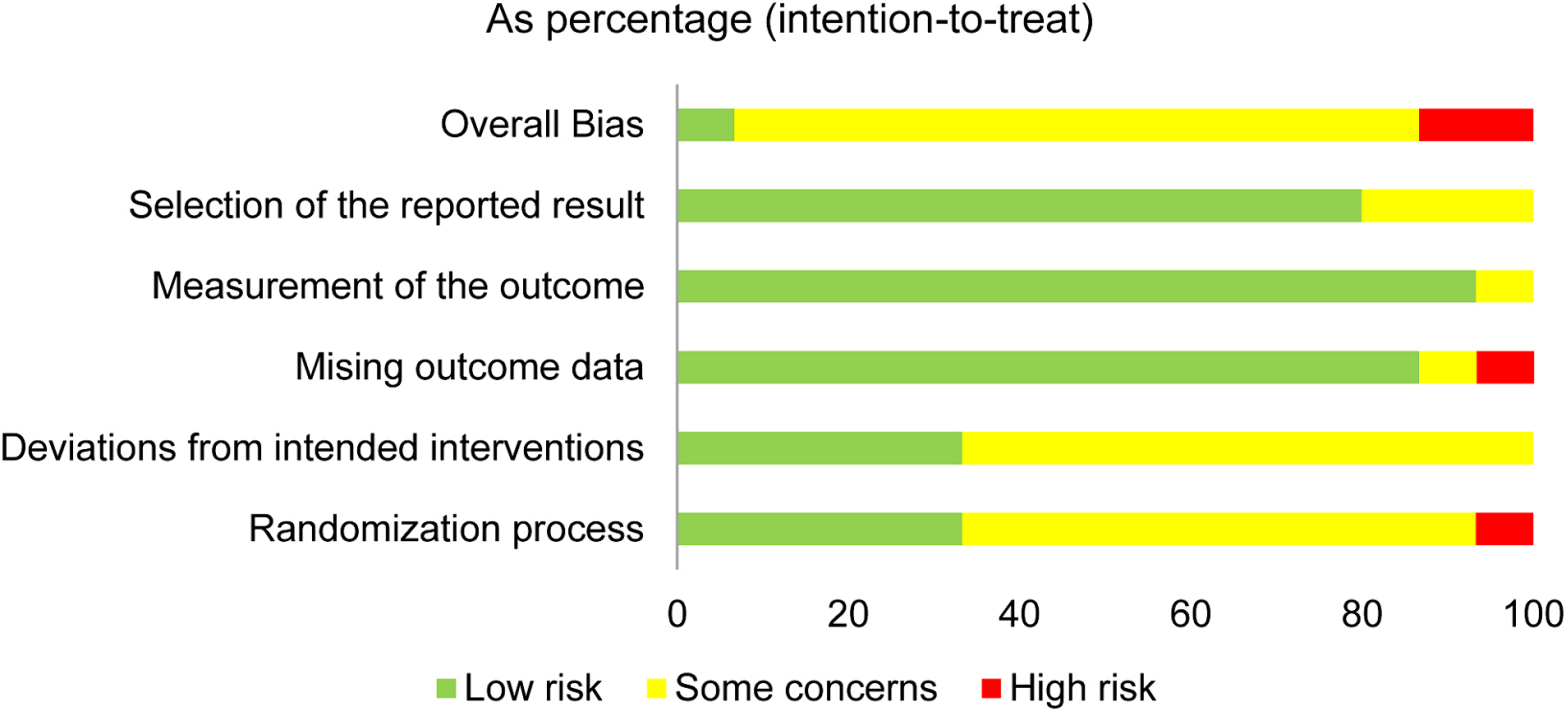
Risk of bias rating within five bias domains 5 as percentage of all trials.

The results of the RoB assessment for the five domains are shown in Figures 2, 3. Arbitration by the third reviewer (FB) was required for one trial. However, overall inter-rater agreement was found to be almost perfect with kappa = 0.84. Only one trial was rated with ‘low risk’ for all five domains (26). The domains missing outcome data, measurement of the outcome and selection of reported results were rated with ‘low risk’ for most trials. In addition, the main domains of concern in the assessment of the trials were the randomization process and deviations from the intended interventions. Only two trials were of high risk for overall bias (25, 33). High drop-out rates during treatment was found in the trial by Limburg et al. (33) (28.4% in experimental group and 52.6% in control group), whereas Gordon et al. (25) did not conceal the allocation.

### 3.3 Trial characteristics including patients with PPPD

All nine trials were identified as randomized controlled trials with two comparators and were published between 2018 and 2023. Included trials were conducted in eight different countries worldwide. The sample size ranged from 19 to 91 patients, with a total of 339 (mean age ranged from 30 to 75 years). Overall, 67% of the patients were female (Table 3).

**Table 3:** Characteristics of included trials with patients with PPPD.

First author/ year of publication/ Country	Number and gender of participants per group	Mean age of participants per group [years]	Dizziness severity/ Symptom duration	Intervention	Comparator	Outcomes	Outcome measures	Training duration/ Training intensity	Results	Drop outs / Flow chart / RoB rating
Choi et al. (23)/ South Korea	VE + OS: 15 (F8, M7) VE: 13 (F8, M5)	VE + OS: 71.5 (Range: 65–78) VE: 75.0 (Range: 67–78)	Moderate/ NR	# Vestibular exercise using a virtual reality system + optokinetic stimulation (VE + OS)	# Vestibular exercise using a virtual reality system (VE)	(1) Impairments caused by dizziness (2) Quality of life (3) Intensity of dizziness (4) Level of anxiety (5) Gait function (6) Postural sway path	(1) DHI (2) ADL (3) VVAS (4) Beck’s anxiety index (5) TUG (6) Dynamic posturography (DP)	4 weeks/ 1 session /week, 20 Min.	**Change from baseline/ median** **VE + OSVE** DHI (−6 pt)DHI (−16 pt)* ADL (−2 pt)*ADL (−15 pt)* VVAS (0 pt)VVAS (−12 pt)* Beck’s (−1 pt)Beck’s (−5 pt)* TUG (− 2 s)*TUG (−2 s)* DP (+2)DP (+4)* VE sign. Improved in all outcome measures, where VE + OS only sign. Improved ADL (*p* = 0.019) and TUG time (*p* = 0.016).	Drop outs: 2 in VE Flow Chart: yes RoB: Some concerns
Eren et al. (24)/ Germany	nVNS: 10 (F6, M4) SOC:9 (F5, M4)	nVNS: 38.8 (SD ±9.8) SOC: 43.2 (SD ±14.1)	Moderate/ NR	# Vagus nerve stimulation (nVNS) + Standard of care	§ Standard of care (SOC) = psycho-education, physical activity, relaxation exercises	(1) Quality of life (QoL) (2) Severity of depression and anxiety (3) Duration of dizziness (4) Frequency of dizziness (5) Severity of dizziness	(1) EQ-5D (2) HADS (3) The duration of the attacks (in minutes) (4) The frequency of the attacks (per week) (5) The severity of dizziness attacks/acute exacerbations as measured by NAS	4 weeks/ nVNS twice daily NR for SOC	**Change from baseline/ mean** **nVNSSOC** QoL (+12.9 pt)*QoL (−5.9) HADS A (−1.2 pt)HADS A (−1.1 pt) HADS D (−2.2 pt)*HADS D (−0.3 pt) Duration (−2.8Min) Duration (−1.7Min) Frequency (+2.4 attacks per week)Frequency (−0.4 attacks per week) Severity (−0.25 pt) Severity (−0.13 pt) Sign. improved quality of life (*p* = 0.04) and HADS depression (*p* = 0.002) only for nVNS.	Drop outs: 4 (2 in SOC and 2 in nVNS) Flow Chart: yes RoB: Some concerns
Gordon et al. (25)/ Israel	AS: 14 (F10, M4) Sham:8 (F6, M2)	AS: 53.1 (SD ±13.3) Sham: 46 (SD ±19.4)	Moderate/ NR	# Active specs (AS) (eyeglasses with referential markers fixed on the lenses)	# Sham specs (markers were placed in peripheral zones, which do not have impact on dizziness)	(1) Impairments caused by dizziness (2) Intensity of dizziness (3) Severity of anxiety (4) Balance	(1) DHI (2) VVAS (3) BAI (4) ABC and BBS	4 weeks/ every day the number of hours per day NR	**Change from baseline/ mean** **ASSham** DHI (−17.7 pt)*DHI (−11.3 pt) VVAS (−2.7 pt)VVAS (−2.1 pt) BAI (−7.8 pt)BAI (−9.4 pt) ABC (+9.7 pt)ABC (+6.5 pt) BBS (+1)BBS (+0.5) Sign. improved DHI for AS (p = 0.04). No group differences regarding ABC, VVAS, BBS and BAI.	Drop outs: 4 (2 in Sham and 2 in AS) Flow Chart: yes RoB: High risk
Herdman et al. (26)/ United Kingdom	INVEST: 20 (F16, M4) VRT: 20 (F16, M4)	INVEST: 44.6 (SD ±17.0) VRT: 44.3 (SD ±17.4)	Severe/ INVEST = 24 months; VRT = 21 months	# INVEST intervention = CBT informed vestibular reha.	# Standard vestibular reha.(VRT)	(1) Impairments caused by dizziness (2) Intensity of dizziness (3) Quality of life (4) Dizziness specific illness perception (5) Cognitive and behavioral responses to dizziness (6) Severity of anxiety and depression	(1) DHI (2) VVAS (3) EQ-5D (4) B-IPQ (5) CBRQ (6) Anxiety with GAD-7, depression with PHQ-9, combined anxiety and depression with PHQ-ADS	4 months/ 6 sessions. The initial session was 60 min, follow-up sessions were 30 min	**Change from baseline/ mean** **INVESTVRT** DHI (−26.6 pt)DHI (−16.3 pt) VVAS (−23.9 pt)VVAS (−16.1 pt) EQ-5D (+0.2 pt)EQ-5D (+0.1 pt) B-IPQ (−22.9 pt)B-IPQ (−11.2 pt) CBRQ (−2.0 pt. toCBRQ (−1.4 pt. to−6.6 pt)−3.8 pt) GAD-7 (−2.6 pt)GAD-7 (−2.1 pt) PHQ-9 (−4.9 pt)PHQ-9 (−2.9 pt) PHQ-ADS (−7.6 pt)PHQ-ADS (−4.9 pt) Small to moderate effects in all measures outcomes in favor of INVEST (SMD = 0.23–0.77).	Drop outs: 6 (3 in INVEST and 3 in VRT) Flow Chart: yes RoB: Low risk
Im et al. (31)/ South Korea	tDCS: 12 (F8, M4) Sham: 11 (F7, M4)	tDCS: 47.8 (SD ±13.0) Sham: 51.7 (SD ±13.1)	Moderate/ tDCS = 17.6 months; Sham = 14.8 months	# tDCS= Transcranial Direct Current Stimulation	# Sham = the current was ramped up to 2 mA over 30 s and ramped down over the next 30s	(1) Impairments caused by dizziness (2) Balance Confidence (3) Severity of anxiety and depression	(1) DHI (2) ABC (3) HARS and HDRS	3 weeks, 15 sessions, 20 min for tDCS, and only 60 s for sham	**Change from baseline/ mean** **tDCSSham** DHI (−5.2 pt)DHI (−9.6 pt) ABC (−5.3 pt)ABC (4.2 pt) HARS (−0.7 pt)HARS (−0.8 pt) HDRS (−0.3 pt)HDRS (−1.5 pt) No sign. Difference between groups in any outcome (*p* > 0.05).	Drop outs: 1 in Sham group Flow Chart: yes RoB: Low risk
Nada et al. (27)/ Egypt	VRT:30 (F19, M11) CG:30 (F17, M13)	VRT:29.6 (SD ± 8.1) CG:31.1 (SD ± 7.6)	Moderate/ VRT = 3.2 years; CG = 2.8 years	# Customized vestibular reha. (VRT) = gaze stab., walking exercises	# Vestibular reha. + placebo tonics of vitamins (control group)	Impairments caused by dizziness	DHI	6 weeks/ 30 min, every day	**Change from baseline/ mean** **VRTCG** DHI (−21.8 pt)*DHI (−22.1 pt)* Sign. improved DHI in both groups. No sign. Difference between groups (p > 0.05).	Drop outs: 0 Flow Chart: no RoB: Some concerns
Teh et al. (29)/ Malaysia	Bal Ex: 15 (F 9, M6) VRT: 15 (F 10, M5)	Bal Ex: 44.0 (SD ± 10.6) VRT: 48.5 (SD ± 8.7)	Moderate/ Bal Ex and VRT = 3 months to >5 years;	# Bal Ex = home-based vestibular reha.	# Vestibular reha (VRT) (clinical-based)	(1) Impairments caused by dizziness (2) Severity of anxiety, depression and stress (3) Quality of life	(1) DHI (2) DASS (3) EQ-5D	12 weeks, 3x/day. 30 min for VRT. Training duration NR for Bal Ex.	**Change from baseline to 12 weeks/ mean** **Bal ExVRT** DHI (−14.1 pt)*DHI (−18.1 pt)* DASS (−4.3 pt)*DASS (−3.5 pt)* EQ-5D (+11.3 pt)*EQ-5D (+7.3 pt)* Sign. improved DHI, DASS-21 and EQ-5D in both groups. No sign. Difference between groups (*p* > 0.05).	Drop outs: 2 (1 in Bal Ex and 1 in VRT) Flow Chart: yes RoB: Some concerns
Yu et al. (28)/ China	SCBT: 46 (F30, M16) CG:45 (F32, M13)	SCBT:42.7 (SD ± 9.8) CG:42.2 (SD ± 9.6)	Moderate/ CBT = 1.8 years; CG = 1.8 years	Sertraline + # CBT (SCBT)	Only sertraline (control group)	(1) Impairments caused by dizziness (2) Severity of anxiety and depression	(1) DHI (2) HDRS and HARS	SCBT = 8 weeks/ CBT 2×1 hour per week, sertraline 50 mg/day and increase to maximum 200 mg/day in the morning, only 4 weeks CG = sertraline in the morning, 4 weeks, same dosage as in SCBT	**Change score from baseline NR** Sign. improved DHI, HDRS and HARS in both groups. By between- group comparison SCBT improved sign. More in all measures outcomes.	Drop outs: NR Flow Chart: no RoB: Some concerns
Yamaguchi et al. (30)/ Japan	VR: 12 (F9, M3) VRT: 14 (F11, M3)	VR: 58.0 (SD ± 17.1) VRT: 63.5 (SD ± 15.9)	NR	# Virtual reality (VR) using the mediVR KAGURA system + VRT	§ Vestibular reha (VRT)	(1) Impairments caused by dizziness (2) Severity of anxiety and depression	(1) NPQ and DHI (2) HADS	1 week for both groups VR = 100 tasks, 10 min VRT = NR	VR sign. Improved in NPQ (*p* < 0.05), HADS-anxiety (*p* = 0.01). No sign. Improvement in control group for any outcome expect for the NPQ visual stimulation (*p* = 0.02).	Drop outs: 2 in VR group Flow Chart: no RoB: Some concerns

In all trials, only two groups were compared, one intervention versus another intervention. A distinction was not always made between experimental and control groups. Therefore, we used the terms “Intervention” and “Comparator”; Dizziness severity classified by DHI: mild (0–30), moderate (>31–60), and severe (>61–100) handicap (34). * = significant outcome, the bold values; # = for more details about intervention see results, part intervention description; § = no detailed intervention description provided in the original publication; ABC = Activities-Specific Balance Confidence; ADL = Activities of daily life; BBS = Berg Balance Scale; BAI = Beck Anxiety Inventory; B-IPQ = Brief Illness Perception Questionnaire; CBT = cognitive behavioral therapy; CBRQ = Cognitive and behavioral responses to symptoms questionnaire. Range of changes among several domains in the table presented, i.e., for fear avoidance, catastrophizing, damage beliefs, embarrassment avoidance, symptom focussing, all-or-nothing behavior and rest/avoidance behaviour; CG = control group; DASS = Depression Anxiety Stress Scales; DHI = Dizziness Handicap Inventory; EQ-5D = measures the quality of life; anxiety; EHI = Edinburgh Handedness Inventory; F = female; GAD-7 = Generalized Anxiety Disorders-7; HADS = the hospital anxiety and depression scale, HADS A = anxiety scale, HADS D = depression scale; HARS = Hamilton Anxiety Rating Scale; HDRS = Hamilton Depression Rating Scale; M = male; Min = minutes; NAS = Numerical Analogue Scale; NPQ = Niigata PPPD Questionnaire; NR = not reported; PHQ-9 = Patient Health Questionnaire-9; PHQ-ADS = Patient Health Questionnaire Anxiety and Depression Scale; PPPD = persistent postural-perceptual dizziness; pt = points; reha. = rehabilitation; RoB = Risk of Bias; s = seconds; SD = standard deviation; SMD = standardized mean difference; sign. = significant; stab. = stabilization; VVAS = Visual vertigo analogue scale; TUG = Timed up-to-go test.

#### 3.3.1 Interventions and outcomes

For most interventions, patients were exercising over a period of four to 12 weeks in an individual setting. Two trials used an intervention of only 3 weeks or less (30, 31). In one trial the patients received the intervention over 4 months (26). Five out of the nine trials used vestibular exercises (alone or supplemented by CBT) either as the intervention or as comparator (23, 26, 27, 29, 30). In the majority of the trials, the interventions were performed supervised in an outpatients setting (23–25, 28), in two trials in a clinic and home setting (26, 29), and in only one trial the intervention was performed at home only (27). Eight out of the nine trials focused on dizziness handicap, as measured by the Dizziness Handicap Inventory (23, 25–31). Four of the nine trials focused on the intensity or severity of dizziness, measured by the Visual vertigo analogue scale and Numerical Analogue Scale (23–26). Four trials focused on the quality of life, using the Activities of daily life and EQ-5D as outcome measures (23, 24, 26, 29), and only two trials focused on gait and balance ability, measured by the Berg Balance Scale and the Timed up-and-go test (25). Other outcomes measures in the trials focused on mental health (i.e., severity of anxiety and/or depression).

#### 3.3.2 Effect of different interventions by patients with PPPD

In the evaluation of the therapeutic effects of optokinetic training compared to vestibular exercise, only patients in the group, who received vestibular exercise using a virtual reality system significant improved in all outcomes (*p* < 0.001) (23).

**Figure 4:**
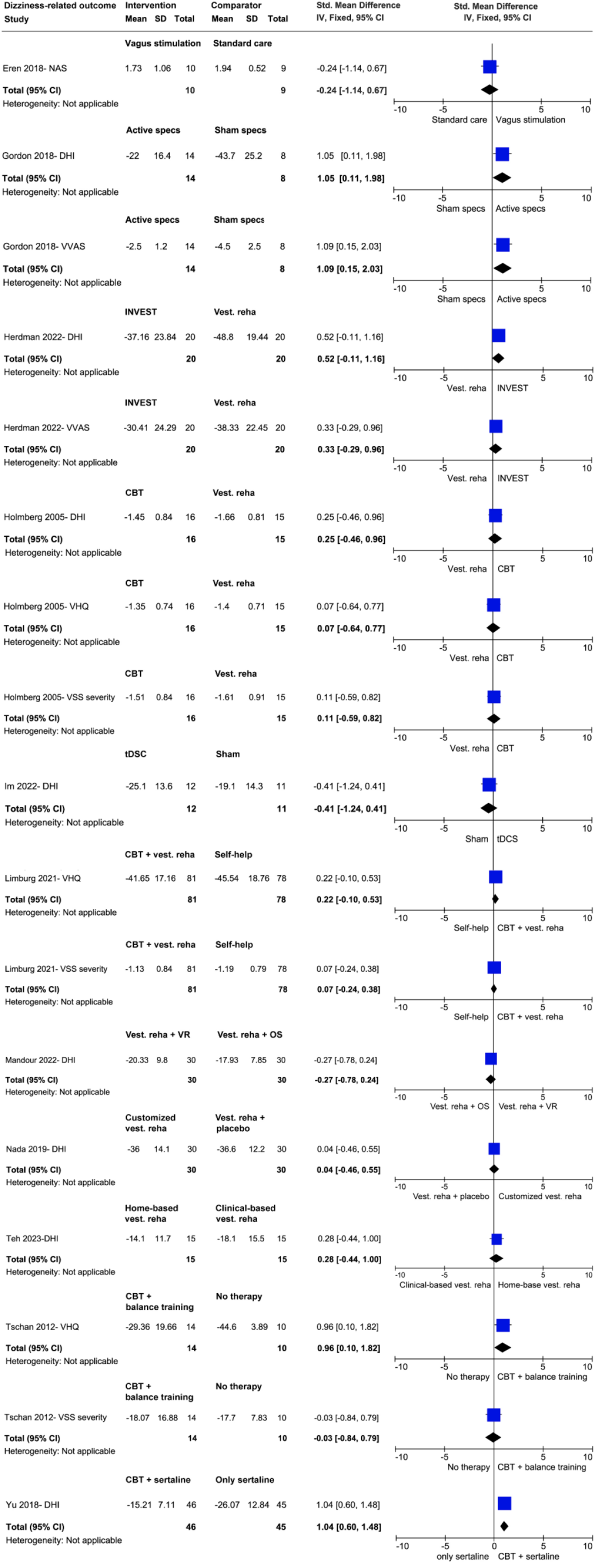
Effect size analyses of different trials regarding dizziness-related outcomes.

**Figure 5:**
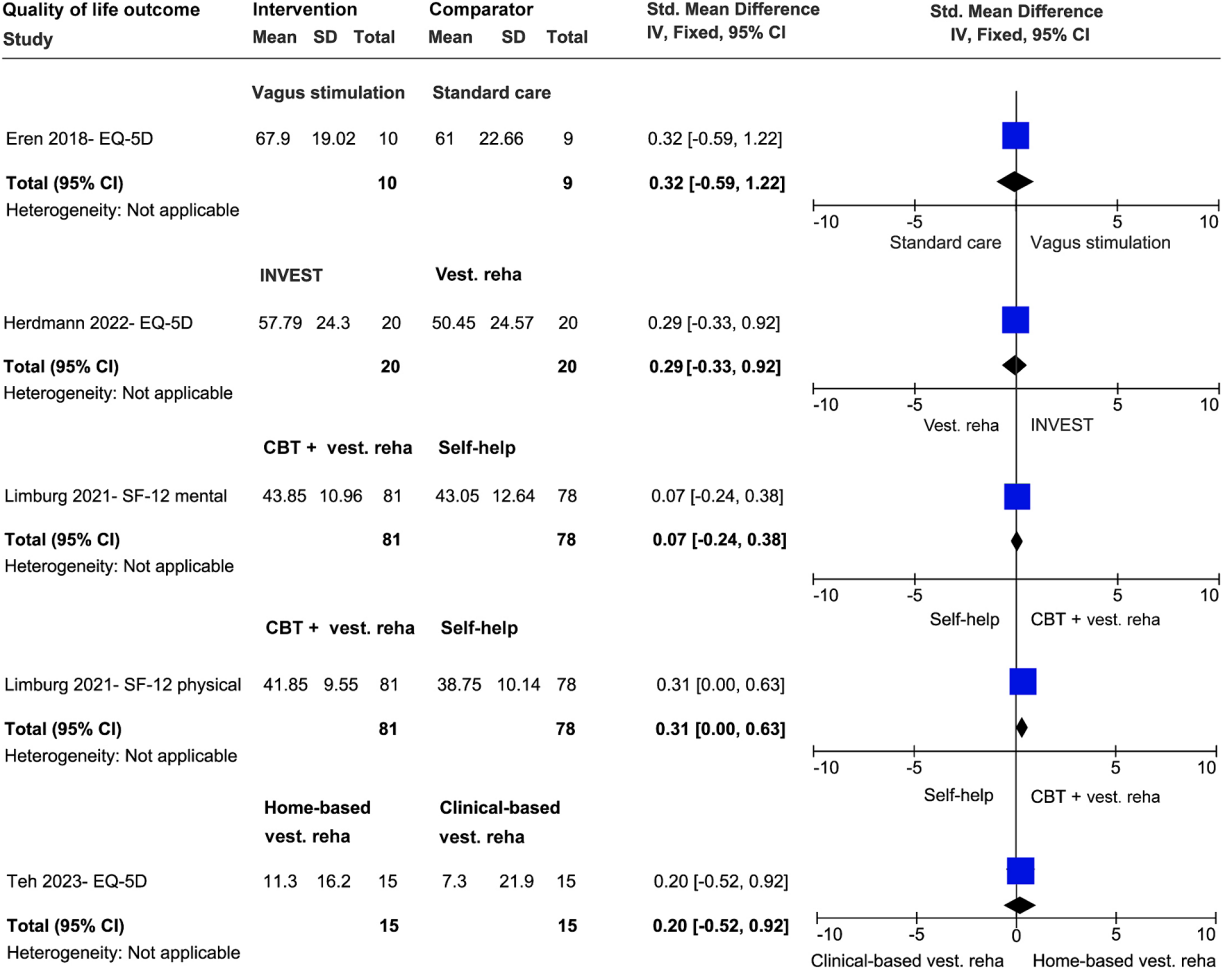
Effect size analyses of different trials regarding quality of life as outcome.

In the comparison between vagus stimulation (VS) and standard care (i.e., psycho-education of the pathophysiology of the PPPD and active and relaxation exercises) on severity of dizziness and quality of life, the weighted SMD was −0.24 (95% CI; −1.14 to 0.67) and 0.32 (95% CI, −0.59 to 1.22), respectively (24). Only the VS group significantly improved in quality of life (*p* = 0.04). No group improved in the outcome severity of dizziness (Figures 4, 5).

**Figure 6:**
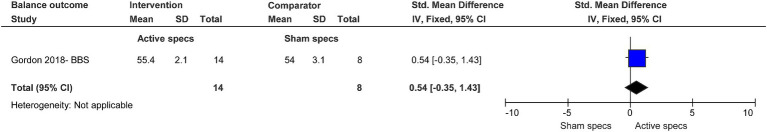
Effect size analysis regarding balance as outcome.

In the evaluation of the therapeutic effects of active specs compared to sham specs on dizziness handicap and severity of dizziness, the weighted SMD was 1.05 (95% CI; 0.11 to 1.98) and 1.09 (95%CI, 0.15 to 2.03), respectively (25). A significant improvement in dizziness handicap showed only the active group (*p* = 0.04). No differences were found regarding balance (Figures 4, 6).

In the comparison between vestibular exercises and vestibular exercises with placebo on dizziness handicap, the weighted SMD was 0.04 (95% CI; −0.46 to 0.55) (27). Both groups improved and no group difference were observed (Figure 4).

In the evaluation of the therapeutic effects of CBT with vestibular exercises (INVEST) compared to vestibular exercise alone, small to moderate effects (SMD = 0.29–0.52, 95% CI, −0.11 to 0.96) for outcomes dizziness handicap, severity of dizziness and quality of life were observed only in INVEST group (Figures 4, 5) (26).

In the evaluation of the therapeutic effects of CBT with sertraline versus sertraline alone on dizziness handicap, the weighted SMD was 1.04 (95% CI; 0.60 to 1.48). Only the group, who received CBT and sertraline significantly improved (Figure 4) (28).

In the evaluation of the therapeutic effects of tDCS versus sham stimulation on dizziness handicap, the weighted SMD was 0.41 (95% CI; −1.24 to 0.41). No group difference was observed (*p* > 0.05) (Figure 4) (31).

In the comparison between the therapeutic effects of home-based vestibular exercises (Bal Ex) versus clinical-based vestibular exercises on dizziness handicap and quality of life, the weighted SMD was 0.28 (95% CI; −0.44 to 1.00) and 0.20 (95% CI; −0.52 to 0.92), respectively (29). No group difference was observed (*p* > 0.05) (Figures 4, 5).

In the comparison between the therapeutic effects of virtual reality using KAGURA-system versus vestibular exercises on dizziness handicap, only virtual reality group improved (*p* < 0.05) (30).

### 3.4 Trial characteristics including patients with functional dizziness

All four trials were identified as randomized controlled trials with two comparators and were published between 2005 and 2022 (32–35). The trials were conducted in three different countries. The sample sizes ranged from 24 to 159 patients, with a total of 279 (age ranged from 18 to 65 years). In total, 129 females and 90 males were included. In one trial, the gender distribution was not reported (35). Please see Table 4 for all details.

**Table 4:** Characteristics of included trials and patient characteristics with functional dizziness (i.e., phobic postural vertigo, visual vertigo and somatoform vertigo).

First author and year of publication / Country	Number and gender of participants per group	Mean age of participants per group [years]	Diagnosis and Dizziness severity + symptom duration	Intervention	Comparator	Outcomes	Outcome measures	Training duration/ Training intensity	Results	Drop outs / Flow chart / RoB rating
Holmberg et al. (32)/ Sweden	CBT: 18 (F9, M 9) PE + VRT:18 (F 9, M 9)	CBT: 40.0 (Range: 23–59) PE + VRT: 47.0 (Range: 30–62)	Phobic postural vertigo/ NR/ CBT = 62 months; PE + VRT= 56 months	#CBT	# Patient education and vestibular reha (PE + VRT)	(1) Impairments caused by dizziness (2) Severity of dizziness and anxiety (3) Severity of depression	(1) DHI and VHQ (2) VSS A, VSS S (3) HADS	NR/ CBT = 45–60 Min/ approx. 10 sessions PE + VRT = 15 Min, 2x/ day	**Change from baseline/ mean** **CBTPE + VRT** DHI (−0.4 pt)* HI (−0.1 pt) VSS A (−0.2 pt)VSS A (−0.3 pt) VSS S (−0.2 pt)VSS S (−0.4 pt)* VHQ (−0.6 pt)*VHQ (−0.2 pt)* HADS A (−0.4 pt)*HADS A (−0.01 pt) **HADS D (−0.3 pt)**HADS D (+0.09) CBT improved sign. in DHI, VHQ and HADS (both scale: depression and anxiety). PE + VRT improved in VSS S (severity scale) and VHQ. Sign. group differences in VHQ and HADS in favor of CBT group.	Drop outs: 5 (2 in CBT, 3 in PE + VRT) Flow Chart: yes RoB: Some concerns
Limburg et al. (33)/ Germany	IPGT: 81 (F49, M 32) SHG:78 (F 49, M 29)	IPGT: 53.7 (SD ± 15.4) SHG: 53.5 (SD ± 15.1)	Functional Vertigo/ Moderate/ ranged from 1 month- over 10 years	# IPGT: CBT + vestibular reha.	§ Self- help group- SHG (patient education without therapeutic intervention)	(1) Impairments caused by dizziness (2) Severity of dizziness and anxiety (3) Severity of depression (4) Somatisation (5) Quality of life	(1) VHQ (2) VSS A, VSS S, BAI (3) BDI (4) PHQ-15 (5) SF-12 P and SF-12 M	16 weeks/ 1 session/ week, 90 min.	**Change from baseline/ mean** **IPGTSHG** VHQ (−15.7 pt)*VHQ (−13.6 pt)* VSS S (−0.2 pt)VSS S (−0.04 pt) VSS A (−0.1 pt)VSS A (−0.1 pt) BAI (−2.5 pt)BAI (−2.3 pt) BDI (−2.3 pt)BDI (−3.4 pt) PHQ-15 (−1.3)PHQ-15 (−1.6 pt) SF-12 P (+1.8 pt)SF-12 P (+2.0 pt) SF-12 M (+3.7 pt)SF-12 M (+1.4 pt) Both groups sign. Improved in VHQ, VSS S, VSS A, BDI and SF-12 M. No sig. Differences between groups for any outcome.	Drop outs: 46 (18 in IPGT, 28 in SHG) Flow Chart: yes RoB: High risk
Mandour et al. (35)/ Egypt	VRT + VR: 30 (sex NR) VRT + OS: 30 (sex NR)	VRT + VR: range 18–65 VRT + OS: range 18–65	Visual vertigo/ Moderate/ VRT + VR = 3.2 years; VRT + OS =2.7 years;	§ Vestibular reha. + Virtual reality (VRT + VR)	§ Vestibular reha. + Optokinetic stimulation (VRT + OS)	(1) Impairments caused by dizziness (2) Visual vestibular mismatch	(1) DHI (2) VVM	4 weeks/ 2 sessions/ week. Session duration VRT + VR = 10 min; OS = at least 30s.	**Change from baseline/ mean** **VRT + VRVRT + OS** DHI (−34.1 pt)*DHI (−33.1 pt)* VVM score NR VVM score NR Both groups sign. Improved in DHI. No sig. Differences between groups.	Drop outs: 0 Flow Chart: no RoB: Some concerns
Tschan et al. (36)/ Germany	CBT: 14 (F7, M7) CG: 10 (F6, M4)	CBT: 52.9 (SD ± 14.1) CG: 47.0 (SD ± 14.3)	Somatoform Vertigo and Dizziness/ Mild/ CBT = 57.1 months; CG = 55.3 months	# CBT+ balance and relaxation exercises	No therapy (control group)	(1) Severity of dizziness and anxiety (2) Impairments caused by dizziness (3) Severity of depression (4) Disease coherence and control over disease	(1) VSS A and VSS S (2) VHQ (3) HADS (4) IPQ-R	9 weeks/ CBT = 1 session/ week, 100 min. CG = no intervention	**Change from baseline/ mean** **CBTCG** VSS A (+0.7 pt)VSS A (−4.9 pt) VSS S (+2.3 pt)VSS S (+1.1) VHQ (−1.8 pt)VHQ (+12.6 pt) HADS A (+0.4 pt)HADS A (+0.6 pt) HADS D (+0.2 pt)HADS D (+0.5 pt) IPQ-R PC (+0.3 pt)IPQ-R PC (−0.4 pt) IPQ-R IC (+0.3 pt)IPQ-R IC (−0.2 pt) CBT improved in IPQ-R (both subscales: personal control and illness coherence). No sign. Improvement in CBT for any other dizziness specific outcomes.	Drop outs: 4 (2 in CBT, 2 in CG) Flow Chart: yes RoB: Some concerns

In all trials, only two groups were compared, one intervention versus another intervention. A distinction was not always made between experimental and control groups. Therefore, we used the terms “Intervention” and “Comparator”; Dizziness severity classified by DHI: mild (0–30), moderate (>31–60), and severe (>61–100) handicap (34) * = significant outcome, the bold values; # = for more details about intervention see results, part intervention description; § = no detailed intervention description provided; approx. = approximately; BAI = Beck Anxiety Inventory; BDI = Beck Depression Inventory; BBS = Berg Balance Scale; CG = control group; CBT = cognitive behavioral therapy; DHI = Dizziness Handicap Inventory; HADS = the hospital anxiety and depression scale, HADS A = anxiety scale, HADS D = depression scale; IPGT = integrative psychotherapeutic group; Min. = minutes; NR = not reported; IPQ-R = Illness Perception Questionnaire-Revised, IPQ-R PC = personal control scale, IPQ-R IC = illness coherence scale; PE + VR = patient education and vestibular rehabilitation; PHQ-15 = Patient health questionnaire; pt = points; reha. = rehabilitation; RoB = Risk of Bias; s = seconds; SD = standard deviation; SHG = Self- help group; SF-12 = Short-Form Health Survey (SF-12 P = physical and SF-12 M = mental scale); VHQ = Vertigo handicap questionnaire; VOR = vestibuloocular reflex; VSS = Vertigo Symptom Scale (two scales: VSS S = severity and VSS A = anxiety); VVM = Visuovestibular mismatch questionnaire.

#### 3.4.1 Interventions and outcomes

In most interventions, patients exercised over a period of four to 16 weeks. One trial did not report the duration of the intervention (32). In two trials the intervention was conducted in a group setting (33, 36). Three out of the four trials investigated the effect of CBT alone or in addition to vestibular exercises (32, 33, 36). In three trials patients performed the intervention supervised in an outpatient setting (32, 33, 36), and in one trial at home and in the clinic (35).

All trials measured dizziness handicap using the Dizziness Handicap Inventory or the Vertigo Handicap Questionnaire. The three trials also focused on the intensity or severity of dizziness, measured by the Vertigo Symptom Scale (two subscales: severity and anxiety) (32, 33, 36). One trial used the SF-12 to evaluate the quality of life (33). In addition, the three trials assessed the severity of anxiety and depression using the Hospital Anxiety and Depression Scale (32, 36), the Beck Anxiety Inventory, and Beck Depression Inventory (33).

#### 3.4.2 Effect of different interventions by patients with functional dizziness

When evaluating the therapeutic effects of CBT compared to patient education added to vestibular exercise on dizziness handicap and severity of dizziness, the weighted SMD was 0.07–0.25 (95% CI; −0.46 to 0.96) (32). Both groups improved in dizziness handicap, but only group, who received the patient education and vestibular exercises improved in severity of dizziness (Figure 4).

When evaluating the therapeutic effects of CBT with vestibular exercises compared to patient education on dizziness handicap and severity of dizziness, the weighted SMD was 0.22 (95% CI; −0.10 to 0.53) and 0.07 (95% CI; −0.24 to 0.38), respectively (33). Both groups improved and no group difference was found. The weighted SMD of therapeutic effects on quality of life was 0.07 and 0.31 (95% CI; −0.00 to 0.63) (Figure 4).

When evaluating the therapeutic effects of virtual reality added to vestibular exercises compared to optokinetic stimulation added to vestibular exercises on dizziness handicap, the weighted SMD was −0.27 (95% CI; −0.78 to 0.24) (35). Both groups improved and no group difference was found (Figure 4).

When evaluating the therapeutic effects of CBT combined with balance and relaxation exercises compared to no therapy on dizziness handicap, the weighted SMD was 0.96 (95% CI; −0.10 to 1.82) and − 0.03 (95% CI; −0.84 to 0.79), respectively (Figure 4) (36).

## 4 Discussion

The aim of this systematic review and the effect size analyses was to provide a comprehensive overview of non-pharmacological treatments and to compare their effectiveness in patients with PPPD. We identified 13 trials, nine of which included clearly described patients with PPPD, and four of which included patients with functional dizziness (i.e., phobic postural vertigo, visual vertigo, somatoform vertigo).

### 4.1 Interventions used in treatment of PPPD and functional dizziness

Trials used different interventions interventions classified into four categories: (1) psychotherapeutic interventions (CBT, patient education), (2) physiotherapeutic interventions/training (vestibular rehabilitation standard or using virtual reality, optokinetic stimulation), (3) stimulation procedures (vagus nerve stimulation, transcranial direct current stimulation) and (4) device application (visual desensitization using personalized glasses, SpotOn Specs). Additionally, a new therapeutic approach, using personalized glasses with neuro balance active markers, were identified and may have a strong effect (SMD = 1.05) on dizziness handicap (25). However, this therapeutic approach should be further investigated in large trials with a longer follow-up period.

Axer et al. (13) proposed a multimodal and interdisciplinary treatment for patients with PPPD, including vestibular rehabilitation exercises combined with CBT and supported by serotonergic medication. In our review, several trials applied vestibular exercises and CBT as single interventions, but only three combined these two therapies strategies together (26, 33, 36). In addition, in one trial, CBT was combined with medication sertraline and compared it with sertraline only (28). The group that received CBT improved significantly more, suggesting that medication only may not be beneficial in treating patients with PPPD. In two trials, the groups receiving vestibular exercises combined with CBT improved more in dizziness related outcomes than the comparing groups (26, 33). Nevertheless, these trials did not report a significant between-group difference. Further randomized controlled trials with large sample sizes are needed.

We classified all identified non-pharmacological interventions into psychotherapeutic interventions, physiotherapeutic interventions/training, stimulation procedures, and therapy with new devices. However, due to the heterogeneity of the trial interventions, we were not able to perform a meta-analysis but we calculated the SMD values and the corresponding 95% CI for each trial and presented as forest plot to visualize the intervention effects for the primary outcomes. The SMD in most trials ranged between 0.04 (small effect) and 0.52 (moderate effect). Only in one trial, the SMD was ≥1.05 (strong effect) (25). However, it is difficult to conclude, which intervention had the greatest effect in the treatment of these patients, as very heterogeneous interventions were compared among each. For example, Choi et al. (23) compared vestibular exercises using virtual reality with vestibular exercises and optokinetic stimulation. They found no effect of optokinetic stimulation on dizziness handicap, which could be interpreted that patients with PPPD but without visual vertigo symptoms have no benefits from an optokinetic stimulation training. However, one possible explanation may also be that realistic backgrounds and active head-eye movements in virtual-reality based vestibular exercises may fully satisfy the habituation process, and additional optokinetic stimuli may be a superfluous. In addition, patients in the optokinetic training group revealed already at baseline a low score on the DHI (34/100) indicating a mild dizziness and therefore less potential for improvement. In the trial by Mandour et al. (35), involving patients with visual vertigo, the same combination of interventions was used, but the optokinetic stimulation was performed without virtual reality. They found a significant improvement in dizziness handicap in both groups. These results could indicate, that patients with visual symptoms benefit from optokinetic stimulation.

### 4.2 Limitations of included trials

For most of the trials, the risk of bias was assessed with some concern for two domains: the randomization process and deviations from the intended interventions. For example, there is a lack of information on whether the order of allocation sequence was concealed until participants were enrolled and allocated to the intervention. Furthermore, no information was provided on whether any deviations from the intended intervention were due to the context of the trial. For only four trials, a trial protocol was published beforehand (23, 25, 26, 33).

Most trials have small sample size (*N* = ≤ 30) and the follow-up period was very short (ranged between 1 week and 3 months after the intervention). Only one trial had a follow-up period of 12 months (33). Eleven out of 13 trials included patients with moderate or mild dizziness. Therefore, is unclear whether patients with severe symptoms equally benefit from the interventions. Therapy contents were described in several trials, but therapy implementation and therapy enhancement was poorly reported.

### 4.3 Strengths and limitations of our review

Our systematic review included 13 RCTs and provides an overview of the non-pharmacological treatments of patients with PPPD and functional dizziness. However, due to the heterogeneity in terms of the interventions and comparators used, we cannot provide final recommendations about the intensity and duration of treatment. However, we observed a large improvement in dizziness-related outcomes (i.e., DHI) in the patient groups that received more than two sessions with 30 min per week for at least 4 weeks. Furthermore, the trials included very heterogeneous groups of patients in terms of anxiety and depression. It is therefore difficult to assess the influence of these psychiatric comorbidities on the persistent dizziness. Further reviews should focus on the outcomes of anxiety and depression and their correlation with dizziness handicap, as these conditions are often associated with the development of PPPD (1, 7, 11, 12).

Moreover, the use of a disease-specific questionnaire of PPPD, i.e., the Niigata PPPD Questionnaire (NPQ) (37, 38), would be beneficial in further trials for assessing the dizziness handicap.

One trial included 18 out of 159 patients (12 in experiment and 6 in control group) with a symptom duration <3 months, who did not meet the criteria for PPPD or functional dizziness (33). Data were presented for all patients together, so it was not possible to consider only data from patients, who had symptoms for more than 3 months. However, as most patients meet the predefined criteria for functional dizziness, we included this trial in our systematic review.

The composition of the search strategy and the search itself were conducted by a professional research librarian from the University of Zurich (CH) in accordance with the review protocol providing a comprehensive search and detailed knowledge of different databases. Moreover, all references were independently selected by two out of three co-authors (of ZS, CZ, and SG). Furthermore, several reviewers extracted and double-checked all the extracted information from the included articles that limited the risk of errors in the extraction process.

## 5 Conclusions and implications for practice and research

The present systematic review and the effect size analyses provides an overview of non-pharmacological interventions for patients with PPPD or functional dizziness, their effects, training intensity and duration. Patients with mild and moderate PPPD or functional dizziness benefit from vestibular rehabilitation, visual desensitization (i.e., eyeglasses with referential marks or optokinetic stimulation), CBT, and vagus nerve stimulation. However, it was not possible to draw final conclusions, which intervention had the greatest effect in the treatment of these patients, as very heterogeneous interventions were compared with each other. More than two sessions per week lasting for 30 min over at least 4 weeks may be more effective. Considering the multifactorial pathophysiology (i.e., excessive vigilance perception of dizziness and imbalance, maladaptive balance control, and visual dependence) and observing the effects of applied intervention among trials, a multimodal approach in the treatment of patients with PPPD is highly recommended.

The multimodal treatment that comprises a combination of vestibular rehabilitation and visual desensitization, CBT including patient education and medication support should be further investigated. Future trials should include a large sample size of patients suffering from severe dizziness, and a longer follow-up period.

## Author contributions

ZS: Conceptualization, Data curation, Formal analysis, Funding acquisition, Investigation, Methodology, Project administration, Resources, Software, Supervision, Validation, Visualization, Writing – original draft, Writing – review & editing. FB: Conceptualization, Methodology, Visualization, Writing – review & editing. CZ: Conceptualization, Data curation, Methodology, Writing – review & editing. SG: Conceptualization, Data curation, Methodology, Writing – review & editing. SS: Conceptualization, Methodology, Writing – review & editing. RH: Conceptualization, Formal analysis, Methodology, Visualization, Writing – review & editing. KP: Conceptualization, Methodology, Writing – review & editing. HG: Conceptualization, Methodology, Writing – review & editing. LB: Conceptualization, Methodology, Writing – review & editing. CS-A: Conceptualization, Methodology, Supervision, Writing – review & editing.
