# Supplementary material for: Comparative effectiveness of non- pharmacological treatments in patients with persistent postural-perceptual dizziness: a systematic review and effect sizes analyses
Source: Front Neurol. 2024 Jul 12;15:1426566. doi: 10.3389/fneur.2024.1426566 (PMC11272556; doi:10.3389/fneur.2024.1426566)
Supplement: Supplementary file 1 [file Table_1.DOCX]

|  | | **Search History Medline** | | |
| --- | --- | --- | --- | --- |
| **Set** | **Search Terms** | | **Limiters/Expanders** | **Results** |
| # 1 | ((MH "Dizziness") OR (MH "Vertigo")) AND (MH "Chronic Disease") OR TI  (((postural-perceptual OR chronic OR persistent OR persisting OR longterm OR "long term" OR longstanding OR somatoform) N3 (dizziness OR vertigo)) OR (postural N3 phob* N3 (dizziness OR vertigo)) OR ((functional OR psycho*) W2 (dizziness OR vertigo)) OR (visual* W1 (induc* OR evoke* OR provoke* OR sensitive OR related) W3 (vertigo OR dizziness)) OR (visual* W1 (vertigo OR dizziness OR dependence)) OR (space N3 motion* N3 discomfort)) OR AB (((postural-perceptual OR chronic OR persistent OR persisting OR longterm OR "long term" OR longstanding OR somatoform) N3 (dizziness OR vertigo)) OR (postural N3 phob* N3 (dizziness OR vertigo)) OR ((functional OR psycho*) W2 (dizziness OR vertigo)) OR (visual* W1 (induc* OR evoke* OR provoke* OR sensitive OR related) W3 (vertigo OR dizziness)) OR (visual* W1 (vertigo OR dizziness OR ependence)) OR (space N3 motion* N3 discomfort)) | | Expanders - Apply  equivalent subjects  Search modes - Find all my search terms | 1,577 |
| # 2 | (PT randomized controlled trial OR PT controlled clinical trial OR TI randomized OR TI randomised OR TI placebo OR TI randomly OR  TI trial OR TI groups OR AB randomized OR AB randomised OR AB placebo  OR AB randomly OR AB trial OR AB groups OR (MW DT)) NOT ((MH animals+) NOT (MH humans)) | | Expanders - Apply  equivalent subjects  Search modes - Find all my search terms | 5,834,846 |
| # 3 | #2 AND #1 | | Limiters -Language:  English, German  Expanders - Apply  equivalent subjects  Search modes - Find all my  search terms | 568 |
